# Interferon Lambda in the Pathogenesis of Inflammatory Bowel Diseases

**DOI:** 10.3389/fimmu.2021.767505

**Published:** 2021-10-12

**Authors:** Jonathan W. Wallace, David A. Constant, Timothy J. Nice

**Affiliations:** Department of Molecular Microbiology and Immunology, Oregon Health & Science University, Portland, OR, United States

**Keywords:** interferon lambda (IFN-λ), intestinal epithelial cell (IEC), inflammatory bowel disease (IBD), ulcerative colitis (UC), Crohn’s disease (CD)

## Abstract

Interferon λ (IFN-λ) is critical for host viral defense at mucosal surfaces and stimulates immunomodulatory signals, acting on epithelial cells and few other cell types due to restricted IFN-λ receptor expression. Epithelial cells of the intestine play a critical role in the pathogenesis of Inflammatory Bowel Disease (IBD), and the related type II interferons (IFN-γ) have been extensively studied in the context of IBD. However, a role for IFN-λ in IBD onset and progression remains unclear. Recent investigations of IFN-λ in IBD are beginning to uncover complex and sometimes opposing actions, including pro-healing roles in colonic epithelial tissues and potentiation of epithelial cell death in the small intestine. Additionally, IFN-λ has been shown to act through non-epithelial cell types, such as neutrophils, to protect against excessive inflammation. In most cases IFN-λ demonstrates an ability to coordinate the host antiviral response without inducing collateral hyperinflammation, suggesting that IFN-λ signaling pathways could be a therapeutic target in IBD. This mini review discusses existing data on the role of IFN-λ in the pathogenesis of inflammatory bowel disease, current gaps in the research, and therapeutic potential of modulating the IFN-λ-stimulated response.

## Introduction

Inflammatory bowel disease (IBD) is the collective term for Ulcerative Colitis (UC) and Crohn’s Disease (CD). IBD has historically been more prevalent in North America, Europe and Oceania, but worldwide incidence is rising, especially in developing nations (1). The Centers for Disease Control recently estimated 3.1 million diagnosed adults in the United States alone (2). Overall prevalence of IBD among Medicare fee-for-service beneficiaries is slightly increasing and the largest increases in incidence are seen in non-Hispanic black persons. IBD and its associated comorbidities can be disabling and patients are often vulnerable to decreasing quality of life (3) as well as significant financial toxicity due to the high cost of therapy (4). Therefore, there remains significant interest in furthering understanding of the mechanisms behind initiation and progression of IBD.

The intestinal epithelium plays a critical role in the pathogenesis of IBD (5), so fundamental understanding of intestinal epithelial cell (IEC) contributions to disease has been of major interest in the field for decades. In the absence of disease, IECs maintain intestinal homeostasis by providing a physical barrier and forming the interface between enteric microbes and the host immune system (6, 7). Disruption of epithelial homeostasis through environmental or infectious triggers can perturb this otherwise peaceful relationship between commensal microbes and the immune system. For example, epithelial barrier breakdown allows excessive exposure to luminal products that promote chronic inflammation and collateral tissue damage and ulceration seen in IBD (8, 9). The chronic inflammatory immune response of IBD results in increased levels of cytokines including tumor necrosis factor alpha (TNFα) and interferons (IFNs).

**Figure 1:**
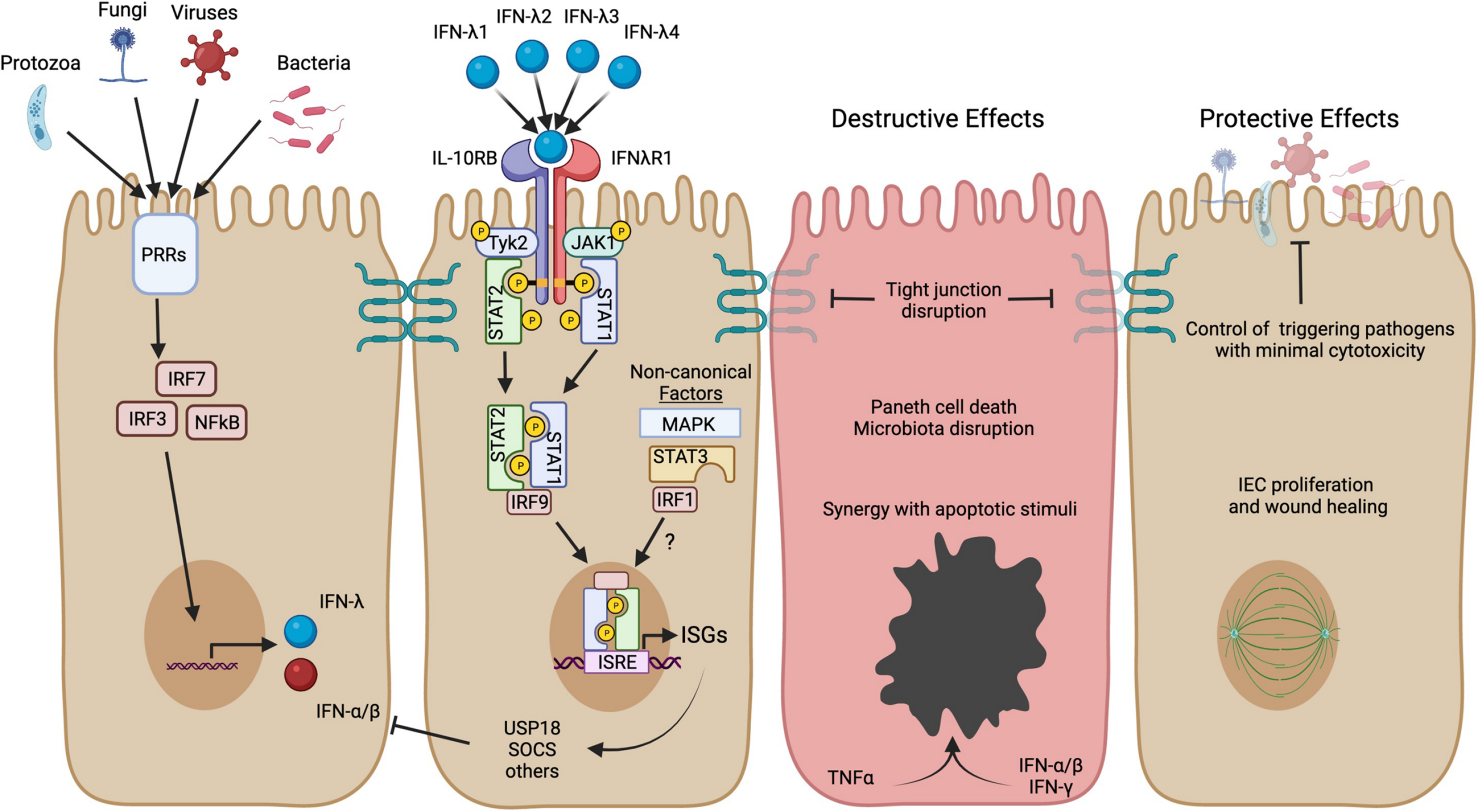
IFN-λ induction, signaling, and potential roles in IBD. Production of IFN-λ can be stimulated by microbial activation of pattern recognition receptors (PRRs) and stimulation of downstream transcription factors (IRF3, IRF7, NFκB). These same transcription factors can also stimulate IFN-α/β, but IECs are able to preferentially produce IFN-λ (19). The four subtypes of human IFN-λ bind to the heterodimeric IFN-λ receptor to activate JAK/STAT signaling and transcription of ISGs. IFN-λ activates the canonical heterotrimeric transcription factor ISGF3 (STAT1, STAT2, and IRF9) to upregulate a core set of ISGs, including direct-acting antiviral effectors and mediators of negative feedback such as *USP18* and *SOCS* genes. Other non-canonical factors such as MAPK, STAT3, and IRF1 can further influence the magnitude and scope of ISGs expressed and may have distinct roles in the context of inflammatory disease. The figure highlights possible detrimental and protective roles for IFN-λ in the context of IBD based on human organoid studies and mouse models of intestinal inflammation (see text for more detail). Created in BioRender.com.

IFNs are named for their important role in interfering with viral replication (10, 11), but have also been implicated in autoinflammatory diseases (12, 13). There are three types of IFNs, each utilizing its own distinctive receptor. Type I IFN consists of at least 17 functional subtypes, including IFN-α subtypes and IFN-β (simplified herein as IFN-α/β) (14). Type II IFN (or IFN-γ) is a single signaling protein that has been of particular interest in studies of IBD. Type III IFN is the most recently identified IFN type and consists of 3-4 IFN-λ subtypes in humans. IFN-λ responses have many similarities with IFN-α/β, but IFN-λ is more restricted in expression of its receptor and physiological functions (15). The receptor for IFN-λ is primarily localized to epithelial cells and IFN-λ has been shown to play a unique and important role in protection of intestinal epithelial cells from enteric viral infections (16–18). Recently, evidence is emerging about a role for IFN-λ in the pathogenesis of IBD, serving as the impetus for this review. In the following sections, we will introduce IFN-λ signaling, discuss protective actions of IFN-λ, and consider contexts where its protective role may not be preserved (**Figure 1**).

## IFN-λ Signaling

Three human IFN-λs (originally named IL-28A, IL-28B and IL-29; later renamed IFN-λ2, IFN-λ3, IFN-λ1, respectively) were discovered in 2003 (20, 21) and a fourth IFN-λ gene (IFN-λ4) was discovered in 2013 (22). The four IFN-λ genes in humans may have non-overlapping roles in UC and CD because studies in other contexts revealed that subtypes of IFN-λ are not functionally equivalent. For example, IFN-λ4 is thought of as having an anti-inflammatory effect due to observations that it is not induced strongly by viral infections like the other IFN-λ subtypes (23) and a disruptive SNP in IFNL4 is associated with clearance of Hepatitis C (22, 24). Comparative transcriptomic studies in hepatocytes suggested minimal differences between IFN-λ1-3 (25), but differing receptor affinity or receptor abundance could significantly impact the scope of a downstream response (26–28). Additionally, differences in the transcription factor usage within the promoter for IFN-λ1 relative to promoter for IFN-λ2 or IFN-λ3 (29) may influence subtype-specific gene expression. Mouse models have been essential to our basic understanding of IFN biology, but it is notable that mice only have two functional IFN-λ genes (IFN-λ2 and IFN-λ3), with IFN-λ1 being a pseudogene and IFN-λ4 being absent (30). Potential differences between subtypes are important considerations for interpretation of results from mouse models and will be interesting to consider in future studies of a role in human disease.

The IFN-λ receptor (IFNLR) is a heterodimeric complex composed of IL10RB and IFNLR1 subunits. IFNLR1 provides specificity for IFN-λ while IL10RB is shared by other IL-10-family cytokines with anti-inflammatory and wound healing functions (IL-10, IL-22, and IL-26) (20, 21, 27). Canonically, the IFNLR1 subunit is associated with Janus-kinase 1 (JAK1) and the IL10RB subunit is associated with the related tyrosine kinase 2 (TYK2) (31). Upon IFN-λ binding to IFNLR, JAK1 and TYK2 phosphorylate signal transducer and activator of transcription 1 (STAT1) and STAT2, which combine with interferon regulatory factor 9 (IRF9) to form a heterotrimeric transcription factor (interferon-stimulated gene factor 3; ISGF3). ISGF3 activates transcription of hundreds of interferon-stimulated genes (ISGs) through binding IFN-stimulated response elements (ISREs) in promoter regions (10).

In the context of complex inflammatory disease such as IBD, multiple cytokine pathways and transcription factors may act in concert (32), with synergy or antagonism in the downstream responses. For IFN family cytokines, homologous interferon regulatory factors (IRF1-IRF9) play combinatorial roles in defining the transcription of IFN-λ, IFN-α/β, and ISGs (33, 34). For example, increased expression of IRF1 can potentiate inflammatory responses to IFN (26) and increased IRF1 has been associated with IBD by expression studies and genome-wide association studies (GWAS) (35, 36). Additionally, JAK and STAT homologs (4 JAKs and 7 STATs) are responsible for an immense complexity of signaling function for a diverse array of cytokines. The complexity relates to cell type specificity, duration, and intensity of STAT signaling (37). The contributions of JAK/STAT signaling to IBD is an active area of study in the field, and is critical to understand in relation to use of existing JAK/STAT inhibitors as therapeutics.

It is clear that the IFN-STAT axis contributes to CD, and recent studies suggest non-redundant contributions of STAT1 and STAT2 in a mouse model of disease (38). STAT3 is also a known gene susceptibility locus for IBD (39, 40), and can be a target of IFN-λ receptor activation in addition to the canonical STAT1/STAT2 response (20, 41). STAT3 was phosphorylated to a low extent by IFN-λ3, but not by IFN-λ1 in one study of the Huh-7 hepatocyte cell line (42), further suggesting the potential for subtype-specific differences. Induction of STAT3 by IFN-λ or IFN-α/β subtypes could be increased in the context of disease and potentiate other proinflammatory cytokines that utilize STAT3 such as IL-6. The role of STAT3 in IBD is likely to be complex with different functions in distinct cell types (43). For example, STAT3 can drive pro-inflammatory effects in Th-17 lymphocytes through IL-23, but STAT3 in IECs has been associated with tissue healing through IL-22 (44). Furthermore, STAT3 can negatively regulate STAT1 signaling *via* sequestration of STAT1 monomers (45, 46). Thus, it is important to consider the contribution of non-canonical signals such as STAT3 when studying the role of IFN-λ in disease.

In addition to the JAK/STAT pathway, IFN-λ also stimulates mitogen-activated protein kinase (MAPK) pathways (47). In contrast to IFN-λ, IFN-α/β-stimulated antiviral protection of IECs does not depend on MAPK (48). In addition to epithelial cells, IFN-λ was found to activate the MAPK pathway in human fibroblasts, which may promote tissue healing *via* upregulation of collagens (49). However, this pro-healing effect may not persist during chronic inflammation where fibrosis is a major complication. MAPK signaling may also be stimulated downstream of TNFα to reduce cell proliferation and increase apoptosis (50), and it would be of interest to study the interactions with IFN-λ in this process. Future studies may further our understanding of the potential for combinatorial MAPK stimulation to be either beneficial in restoring homeostasis or maladaptive in the chronic inflammatory landscape of IBD.

## Evidence for a Protective Role of IFN-λ in IBD

Biopsies from IBD patients show increased expression of both IFN-λ and IFNLR in the intestinal mucosa, with no discernable differences between CD and UC (51). However, these associations do not indicate whether IFN-λ plays a protective or detrimental role. In fact, distinct roles may manifest in different anatomical locations exemplified by one study showing overexpression of IFN-λ contributes to Paneth cell death in the mouse small intestine (52) and a separate study showing that IFN-λ improves mucosal healing in colonic tissue using a knockout mouse model (51). In this section we will consider the evidence for protective roles of IFN-λ, with evidence for detrimental roles considered in the next section.

IFNLR knockout mice exhibited increased tissue inflammation following dextran sulfate sodium (DSS)-induced colitis relative to WT controls, suggesting that induction of IFN-λ could be tissue protective in this acute inflammation model (51). The authors show that the IFN-λ response promotes mucosal healing and increases IEC proliferation, with associated increases in expression of specific ISGs such as *USP18*, which has been shown to desensitize cells to the IFN-α/β response (53, 54). Additional studies in IFNLR knockout mice have corroborated findings that IFNLR promotes mucosal healing in the colon. When IFNLR was knocked out alone or in IFNLR/IFNAR double knockouts, DSS colitis susceptibility increased compared with controls (55, 56). In double receptor knockouts, slowed recovery from DSS colitis was associated with impaired IEC proliferation and loss of goblet cells with mucin granules (55). Increased infiltration of neutrophils and CD169+ macrophages were also observed in double receptor knockouts, but interestingly, larger increases occurred in mice deficient in only the IFNLR receptor. Bone marrow chimeras demonstrated that IFN-α/β exerts its effects primarily haematopoietically whereas IFN-λ exerts its effect primarily from within the colonic epithelium (55). However, separate studies have implicated IFN-λ in protection from intestinal inflammation by acting haematopoietically. IFN-λ was found to suppress neutrophil generation of reactive oxygen species in a DSS colitis mouse model (57). Other recent studies of intestinal inflammation associated with graft versus host disease reported a partial, protective role for hematopoietic expression of IFN-λ receptor that is distinct from promoting epithelial cell proliferation and mucosal healing (58). Taken together, these studies point to protective mechanisms for IFN-λ in promoting epithelial healing during acute colonic inflammation and suggest additional protective roles of non-epithelial cell types.

There are also indications of a protective role for IFN pathway factors. IRF3 and IRF7 are important for induction of IFN-λ, IFN-α/β, and ISGs; experiments in IRF3/IRF7 double knockout mice with DSS colitis showed greater tissue inflammation relative to controls (51), suggesting a mucosal protective effect of IFN responses in colonic tissues. Toll-like receptor 3 (TLR3) is important in antiviral immunity and can induce IFN-λ as well as IFN-α/β. While the specific patterns of TLR expression by IECs remains a controversial area, one study reported that TLR3 is downregulated in CD but not UC (59). Additionally, polyinosinic-polycytidylic acid (poly-I:C, TLR3 agonist) treatment was shown to protect mice from tissue damage in DSS colitis in a TLR3-dependant manner (60, 61). However, TLR3 stimulation also increases expression of proinflammatory chemokine CXCL10 which is increased in active IBD (62), and thought to promote pathology. Thus, the roles of IFN-λ may be more complex when combined with other IFN family members.

In addition to direct effects on inflamed tissues, IFN-λ may be indirectly protective against IBD *via* defense against pathogenic microbes that could trigger IBD symptom onset or exacerbate flares (63). Norovirus is of particular interest because murine norovirus infection synergizes with deficiency in *Atg16L1* (autophagy- and CD-associated gene) to increase risk of DSS-triggered colitis (64). This interactive phenotype was ameliorated by anti-TNFα, anti-IFN-γ, or antibiotics, implicating similar pathways influential in human IBD. Mouse norovirus infection of IECs is controlled by IFN-λ (17, 65), which would indirectly block inflammation triggered by this virus infection. Furthermore, IFN-λ stimulates antiviral gene expression in IECs, with limited stimulation of proapoptotic genes, thereby minimizing epithelial damage during an inflammatory immune response (66). In addition to antiviral defense, IFN-λ restricts fungal infections such as *Aspergillus fumigatus* (67), protozoal infections such as *Cryptosporidium parvum* (68), and is produced upon TLR stimulation by bacterial products (69). Defense against *Aspergillus* is particularly notable because increased colonization is observed in areas of inflamed UC tissue (70). *Aspergillus* has also been observed to be overabundant in fecal samples of patients with CD compared with healthy subjects (71). In addition to pathogens, it is interesting to speculate that IFN-λ responses may influence the host response to the non-pathogenic microbiota, but definitive studies in this area are still needed. In sum, the anti-pathogen activities of IFN-λ are anticipated to indirectly limit infectious triggers of IBD.

## Evidence for a Destructive Role of IFN-λ in IBD

Changes to the structure and expression of tight junction proteins is known to occur in both CD and UC (72–76). Studies of IEC organoids indicate that barrier permeability is increased by IFN-λ exposure without an increase in apoptosis, but rather associated with disruption of junctional proteins E-cadherin and ZO-1 (77). The barrier disruption caused by IFN-λ in organoids is eliminated by pretreatment with the clinically-relevant JAK1 inhibitor filgotinib (77), consistent with a requirement for this IFNLR1-associated kinase. Disruption of E-cadherin is especially notable because it has been associated with barrier function breakdown in CD (78). Even though IFN-λ may have some disruptive properties, IFN-γ stimulation of IEC organoids more potently disrupted barrier integrity (77), suggesting it may be a more prominent mediator of pathology in IBD. Additionally, IFN-γ inhibits expression of SLC26A3 (79), a chloride bicarbonate ion exchanger which is known to normally protect against TNFα–induced barrier destruction (80). Lower levels of SLC26A3 are associated with intestinal epithelial barrier dysfunction *via* modulation of junctional proteins (81). It is likely that the different IFN types may synergize or inhibit each other in their effect on junctional proteins, an area for future study.

Paneth cell death is an important factor in CD as it causes dysregulation of microbial control at epithelial barriers due to loss of secreted antimicrobial peptides. A recent study associated elevated IFN-λ with increased Paneth cell death in CD compared to controls (52). A mouse model of elevated IFN-λ also revealed decreased Paneth cell numbers and epithelial apoptosis that was restored by STAT1-deficiency (52). Additionally, STAT1 (but not STAT2) deficiency partly restores Paneth cell numbers in the Caspase-8-deficient mouse model of intestinal inflammation (38). A cytotoxic effect may be even stronger for other IFN types because organoid studies have shown increased IEC death following treatment with IFN-β or IFN-γ (66, 77). Even so, the effects of IFN-λ on death of Paneth cells or other IEC types may be amplified by signaling factors such as IRF1. IRF1 was shown to have increased expression in the epithelium of IBD patients (36), and its expression amplifies inflammatory chemokine secretion and cytotoxic responses to TNFα (26, 36). Additionally, IFN-stimulated effects may be further amplified by deficiency in autophagy, as indicated by discovery of a role for autophagy gene ATG16L1 in preventing TNFα-driven necroptosis of Paneth cells (82). In sum, excessive death of IECs is a critical driver of intestinal mucosa disruption during IBD and further study is needed regarding the role that IFN stimulation plays in combination with other signals such as TNFα or autophagy.

## Therapeutic Potential of the IFN-λ Pathway

Polyethylene glycol (PEG)-conjugated IFN-λ therapy has been examined for viral infections, including Hepatitis C, Hepatitis B, and recently SARS-CoV-2. PEG-IFN-λ has not been tested as a therapy for IBD. However, IFN-α/β has been tested in multiple trials, but there was no improvement compared to placebo and generally low tolerability with increased withdrawal due to adverse events (83). These results are consistent with the generally low tolerability of therapy. Although mouse models of UC show evidence of a pro-healing role for IFN-λ, any potential for PEG-IFN-λ therapy would need to identify a critical balance between its helpful and detrimental roles such as its potential to cause Paneth cell death. On the other hand, IFN-λ neutralization might be an option based on potential for detrimental effects in CD. However, there has been more interest in neutralization of IFN-γ because of its more robust role in driving IBD pathology. Although neutralization of IFN-γ has shown efficacy in mouse models (64), anti-IFN-γ (fontolizumab) trials for CD showed poor clinical efficacy and were discontinued (84), but a combined inhibition across IFN types or together with anti-TNFα may be worthy of consideration.

The JAK/STAT pathway is an attractive therapeutic option based on the potential synergistic effects across IFNs and other cytokine networks, and has currently approved inhibitors and several investigational products. JAK inhibitors may have differential efficacy in IBD subsets due to evidence for differential regulation in CD vs UC (39). Tofacitinib, an inhibitor of JAK1-3, was found to be efficacious in UC trials (85), but not in trials for treatment of CD (86). In contrast, a selective inhibitor of JAK1 (filglotinib) was found to increase remission of CD (87), and other trials are underway with a different JAK1 selective agent, upadacitinib (88). Due to the central role of JAK1 in the IFN response, the necessity of JAK1 selective inhibition in CD is supportive of a pathogenic role for IFN signaling in this disease subset.

In addition to therapeutic modulation of IFN signaling, IFN-λ or ISGs may be useful biomarkers to predict responsiveness to anti-TNFα treatments. The heterogeneity in therapeutic responsiveness and the significant systemic adverse effects associated with anti-TNFα therapy have compelled an extensive search for predictive biomarkers (89). Although anti-TNFα is among the best available treatment options, approximately one third of patients do not respond (90). ISG signatures prior to treatment have been shown to be lower in peripheral blood of patients who responded to anti-TNFα therapy (91), suggesting that increased IFN response can limit therapeutic efficacy. IFN-λ is elevated in serum during active CD, but showed significant heterogeneity among patients (52, 77). Thus, it would be interesting to determine whether IFN-λ, other IFN types, or specific sets of ISGs are superior biomarkers for prediction of anti-TNFα responsiveness.

## Conclusion

IFN-λ is critical for immunological function and barrier integrity at mucosal surfaces. Control of pathogenic microbes at the intestinal epithelium without inducing overly robust inflammatory responses is a well-established normal function of IFN-λ. Additionally, there is evidence that IFN-λ promotes healing in colonic epithelial tissues and *via* ISG expression in IECs themselves as well as *via* regulation of other inflammatory cell types such as neutrophils. However, a maladaptive role might be assumed in the setting of chronic inflammation. In particular, studies of Paneth cells in human subjects and mouse models suggest that IFN-λ takes a detrimental rather than protective role in the small intestine in CD. Finally, IFN-λ as a biomarker for prediction of response to anti-TNFα is an intriguing area for investigation, and continued consideration of JAK inhibitors that act across IFN types will be important.

## Author Contributions

JW wrote the initial draft and all authors revised, edited, and approved the final manuscript. All authors contributed to the article and approved the submitted version.

## Funding

DC was supported by NIH grant T32-AI007472 and the Medical Research Foundation of Oregon (OHSU). TN was supported by NIH grant R01-AI130055. The funders had no role in study design, data collection and interpretation, or the decision to submit the work for publication.

## Conflict of Interest

The authors declare that the research was conducted in the absence of any commercial or financial relationships that could be construed as a potential conflict of interest.

## Publisher’s Note

All claims expressed in this article are solely those of the authors and do not necessarily represent those of their affiliated organizations, or those of the publisher, the editors and the reviewers. Any product that may be evaluated in this article, or claim that may be made by its manufacturer, is not guaranteed or endorsed by the publisher.
